# Anticipation of High-Sensitivity C-Reactive Protein Effect on Post Myocardial Infarction Depression Disorder

**DOI:** 10.31661/gmj.v10i0.1512

**Published:** 2021-04-28

**Authors:** Parichehr Alizadeh, Ehsan Bahramali, Arvin Hedayati, Azizallah Dehghan

**Affiliations:** ^1^Student Research Committee, Fasa University of Medical Sciences, Fasa, Iran; ^2^Noncommunicable Diseases Research Centers, Fasa University of Medical Sciences, Fasa, Iran; ^3^Research Center for Psychiatry and Behavioral Sciences, Shiraz University of Medical Sciences, Shiraz, Iran

**Keywords:** Myocardial Infarction, Depression, Hs-CRP, Atherosclerosis

## Abstract

**Background:**

The natural history of acute myocardial infarction (AMI) as the most prevalent public health issue in Iran has changed with the introduction of novel therapeutic strategies that have reduced its mortality significantly. Major depressive disorder (MDD) is a prevalent and disabling psychiatric disorder and frequently co-exist with AMI. There are proposed pathophysiological links between the two diseases among which inflammation is the most important. With more patients surviving a myocardial infarction (MI) event, post-MI depression has become an important determinant of disability and mortality.

**Materials and Methods:**

In this study we defined a 1-month post-MI depressive scale of 200 patients using Beck’s inventory questionnaire II and measured serum high Sensitivity C-Reactive Protein (hs-CRP) and carotid intima-media thickness (CIMT) to look for the association between inflammatory state and atherosclerosis in different depression score categories.

**Results:**

Minimum and maximum Beck scores were 1 and 43, respectively with a mean of 13±8. The mean CIMT was 0.77±0.26 mm. Serum hs-CRP level was measured with a mean of 1.51±1.6 mg/L. According to BDI-II scores, 44.2% of patients 1-month post-MI suffered from more than mild depression. Being affected was not correlated with either the level of hs-CRP or CIMT. Nearly 44 percent of patients suffered more than mild depression. There was a negative association between serum hs-CRP level and CIMT as a measure of atherosclerosis in groups of depressed versus non-depressed patients. This may indicate that the extent of atherosclerosis is not correlated with the inflammatory state after MI in depressed versus non-depressed patients.

**Conclusion:**

The results of this study indicate that the extent of atherosclerosis is not correlated with the inflammatory state after MI in depressed versus non-depressed patients. Nonetheless, the prognostic indications of increased hs-CRP and depression after AMI remains to be investigated further.

## Introduction

Cardiovascular diseases (CVD), mainly coronary artery disease (CAD), including acute myocardial infarction (MI), are the most common reasons for mortality worldwide and imposing economic costs in developed countries [1]. In the last four decades, the mortality rate has declined in North America and Western Europe due to primary prevention measures besides the deployment of appropriate treatment strategies, and as a result, the quality of life of post-MI has become more important [2]. In Iran, acute MI, the most common cause of death, affects patients 10 years earlier than in developed countries [3]. Its in-hospital mortality was 12.1%, which was higher in women than in men [4]. Hypertension, smoking, and dyslipidemia (DLP) were the three major conventional risk factors for acute MI in Iran [5]. Major depression disorder (MDD) ranked 4th in the list of diseases causing disability worldwide, with the projections revealing MDD to become the 2nd in 2020 [6]. There is a high comorbidity between CVD and depression, which is probably bidirectional [7]. Many studies show that one week after acute MI, up to 50% of patients suffer from anxiety and/or depression symptoms. This adds to the morbidity of acute MI because post-MI patients with anxiety have higher rates of hospitalization. Furthermore, researchers indicated that post-MI depressive symptoms lead to increased mortality rates [8]. It is also reported that the suicide risk is at its highest during the first month after discharge, even with no history of psychiatric illness [9]. The prevalence of depression following acute MI has been reported to be between 45% to 90% in Iran [10]. Whereas these reports seem to be exaggerated, there is no doubt that the prevalence of depression after acute MI is significantly higher than a healthy population, portending the necessity for better psychiatric care to those suffering from acute MI [11]. Interventions, including primary angioplasty, have dramatically reduced AMI mortality and cardiovascular (CV) morbidity, and a larger number of patients survive an acute MI event. However, comorbidities, including depression, play a major role in the prognosis and quality of life [12]. As we know, elevated levels of proinflammatory cytokines, including C-reactive protein (CRP) and interleukin-6 (IL-6), are associated with increased CV morbidity and mortality [13]. Also, these cytokines have been linked to the pathophysiology of atherosclerosis and diseases like depression [14]. These findings are suggestive of s common inflammatory background for both diseases and may have prognostic indications after acute MI. This study was conducted to anticipate of high-sensitivity-CRP (hs-CRP) effect on post-MI depression disorder.

## Materials and Methods

###  Patients and Study Population

 In a cross-sectional study design, we sampled 200 patients who had suffered from an MI event at a University Hospital in the Fasa city, Sothern of Iran, via convenience method. We enjoyed the infrastructure which was already available as the population-based registry for MI in the city of Fasa called Fasa Registry for MI (FaRMI) to select and recruit our patients consecutively [15]. We aimed to look for the association between hs- CRP and the prevalence of depressive mood post-MI in patients with a variety of extent of atherosclerosis in arteries, measured as the carotid intima-media thickness (CIMT).

###  Data collection

 A total of 200 patients who suffered from a first episode MI were recruited. We used the World Health Organization definition [16] of MI to include patients in the study, which requires at least two of the following three criteria for the diagnosis of acute MI; typical chest pain, characteristic electrocardiographic changes, and a serial rise in serum biomarker levels of MI. We exclude those with chronic infection or other inflammatory diseases, who suffered from a significant psychiatric illness other than depressive symptoms or received antidepressants or other psychotropics, with a previous history of MI, who were medically unstable to complete the interview, and older than 80 years of age. After one month of the index MI event, we interviewed patients and filled out questionnaires about their physical health and use of drugs. A trained nurse, per standard protocols, assessed their anthropometric measurements, including height, weight, and vital signs. We also enjoyed the established disease registry system for MI in the hospital, which has systematically recorded the demographic data as well as the marital and socioeconomic status beside conventional risk factors profile of each individual, including a history of and the incident hypertension, diabetes, hyperlipidemia, and cigarette/ hookah/opium consumption of patients.

###  Depression Measurement

 Measurements of depressive symptoms were obtained using the Beck depression inventory II Scale (BDI-II). This form has 21 items to measure the presence and severity of depressive symptoms. Each item is scored on a 4-point scale ranging from 0 to 3. The total score ranges from 0 to 63, with higher scores indicating greater levels of depressive symptoms. Scores≤13 indicate minimal depressive symptoms, scores of 14-19 indicate mild depressive symptoms, scores of 20-28 indicate moderate depressive symptoms, and scores≥29 indicate severe depressive symptoms We used the persian version of BDI-II which has been proved valid and reliable and Cronbach's alpha of this questionnaire was reported to be 0.87 [17].

###  hs-CRP Measurement

 Serum sample was used to measure hs-CRP and the basis of the turbidometric method (immune turbidometery) is based on the formation of a complex resulting from the reaction between hs-CRP and its specific antiserum.

###  CIMT Measurement

 CIMT measurement was performed by noninvasive ultrasound method. In this study, Mylab70 Xvision and linear transducer with a frequency of 13 MHz were used. Patients were initially supine and their heads rotated 45 degrees to the front of the study area. Longitudinal and transverse scans were performed in B-Mode with transducer motion to identify the areas with the greatest thickness in the bulb area (one centimeter proximal to the common carotid bifurcation) of both carotids. A high quality image was taken of the target area. The image was then fixed and the measurement was performed electronically by placing a marker. CIMT was measured in the distal common carotid artery and proximal to the internal carotid artery and bulb at three points on the right and left. The mean of the measurements was recorded as the mean of CIMT.

###  Ethical Considerations

 Written informed consent was taken from all participants and the study was approved by the ethics committee at Fasa University of Medical Sciences (approval code: IR.FUMS. REC.1397.148).

###  Statistical Analysis

 Independent sample t-test and Pearson correlation test was used for analysis. All analyzes were performed using SPSS 22 (SPSS Inc., Chicago, Illinois, USA).

## Results

 A total of 154 patients were interviewed, and carotid sonography and hs-CRP measurements were done for them.Interviews were made at the physician in charge’s discrestion and those with unstable hemodynamic conditions were excluded (14 patients). Thity two more patients were not allowed to have the sonographic examination because of completed bed rest or limited out of bed conditions and were also excluded. Minimum and maximum Beck scores were 1 and 43, respectively with a mean of 13±8. According to studies, 86 people (55.8%) in this study had the minimal depressive symptoms and were in the healthy group. Also 32 patients (20.8%) had mild depression, 30 patients (19.5%) had moderate depression and 6 patients (3.9%) had severe depression. The mean CIMT was 0.77 mm (0.49- 2.61±0.26). Serum hs-CRP level was measured with a mean of 1.51 mg/L (0- 8.2±1.6). According to BDI-II scores, 44.2% of patients 1-month post-MI suffered from more than mild depression. Being affected was not correlated with either the level of hs- CRP or CIMT (Table-1) and Table-2).

## Discussion

Post-MI depression is a common condition that can theoretically aggravate patient outcomes and influence the incidence of Major Adverse Cardiovascular Event (MACE) rate [18]. It is not clear whether depression itself or the accompanying conditions like atherosclerosis or inflammation contribute to the worse outcome in this patient population. Here we examined the correlation of being affected with depression one-month post-MI with the overall inflammatory status of the body and the extent of atherosclerosis represented by hs-CRP and CIMT respectively. Our findings couldn’t reveal a positive association that signifies alternative mechanisms involved in the pathophysiology of depression after MI. Determinants of the well-established relationship between depression and MI remained to be investigated more concerning other possible pathophysiological aspects [19]. The guideline-oriented use of anti-platelets, statins, and revascularization strategies has enormously improved outcomes in patients with MI and regardless of the extent of atherosclerosis or inflammatory response of the body, these strategies have helped patients survive and live longer free of further events. Maybe in the era of post-revascularization and use of high dose statins, depression and the molecular mechanisms involved, can only minimally affect future outcomes [20,21]. This is besides the non-compliance for medication that is frequently observed in these patients and can gravely affect the post- MI patient care and minimize the therapeutic efficacy of prescribed medications [22]. A search to find a pathophysiological link between depression, inflammation and atherosclerosis must then include those yet not affected with either of the conditions to make it possible to look for the common pathways leading to the events of MI, stroke, or other atherosclerosis manifestations as well as depression. This may encompass genetic, environmental, and socioeconomic determinants and needs to be addressed in separate future studies [23]. Our study, though reporting a negative association is better comprehended when seen in a wider context; the temporal correlation of MI and depression was not clear and we didn’t know which one was pre-existing. Conceptualization would be different if MI happens in patients with known depression rather than a patient becoming depressed after an MI event. Maybe by this discrimination, we could find a positive association with inflammatory responses in those already being affected with depression. We take this as a limitation of our study and recommend a future study that follows a cohort of patients with depression and records the events of MI, stroke, and death prospectively to investigate the involved mechanisms.

## Conclusion

 Our survey presents a theoretical explanation of how depression, inflammation, and atherosclerosis burden can be correlated in patients with MI and suggests future studies to investigate the possible pathophysiological mechanisms involved as common pathways.

## Acknowledgment

 We would like to thank the Vice Chancellor for Research of Fasa University of Medical Sciences for supporting this project (grand number:97204).

## Conflict of Interest

 None.

**Table 1:** Pearson Correlations Between Score of Depression and Left Carotid Thickness, Right Carotid Thickness, hs-CRP, and Max-Carotid.

**Variables**	**R**	**P-value**
**Left carotid**	0.012	0.886
**Right carotid**	0.02	0.803
**hs-CRP**	0.079	0.332
**Max-Carotid**	-0.001	0.99

**Table 2:** Compare Mean of Ltcarotid, Rtcarotid, Hs-CRP, and Max-Carotid Between Depressed and Healthy People.

**Variables**	**Groups**	**Mean**	**SD**	**P-value**
**Lt carotid**	Healthy (n=86)	0.73	0.28	0.832
	Mild (n=32)	0.73	0.21
	Moderate (n=30)	0.74	0.24
	Major (n=6)	0.75	0.25
**Rt carotid**	Healthy (n=86)	0.67	0.13	0.864
	Mild (n=32)	0.66	0.16
	Moderate (n=30)	0.68	0.15
	Major (n=6)	0.67	0.15
**Hscrp**	Healthy (n=86)	1.45	1.53	0.574
	Mild (n=32)	1.49	1.36
	Moderate (n=30)	1.57	1.72
	Major (n=6)	1.58	1.69
**Max-Carotid**	Healthy (n=86)	0.78	0.27	0.739
	Mild (n=32)	0.76	0.26
	Moderate (n=30)	0.77	0.24
	Major (n=6)	0.77	0.26

**Lt:** Left; **Rt:** Right.
